# Isolated superior rectus-levator complex enlargement: Inflammatory etiology, clinicoradiological features, and treatment outcomes

**DOI:** 10.1371/journal.pone.0338854

**Published:** 2026-09-23

**Authors:** Min Kyu Yang, Seong Jung Ha, Ho-Seok Sa

**Affiliations:** Department of Ophthalmology, Asan Medical Center, Seoul, Republic of Korea; Hangil Eye Hospital / Catholic Kwandong University College of Medicine, KOREA, REPUBLIC OF

## Abstract

**Purpose:**

To analyze the etiology, clinicoradiological features, and treatment outcomes of orbital inflammation presenting with isolated superior rectus-levator complex (SR-LC) enlargement.

**Methods:**

We retrospectively reviewed patients treated for orbital inflammation with isolated SR-LC enlargement, assessing the underlying etiologies, including thyroid eye disease (TED), idiopathic orbital myositis (IOM), and immunoglobulin G4-related ophthalmic disease (IgG4-ROD). The IOM group was further divided by the presence of upper lid ptosis. Clinicoradiological findings and outcomes were compared between the TED and IOM groups.

**Results:**

Among the 58 patients, 39 (67.2%) had TED, 17 (29.3%) had IOM (10 without ptosis and seven with ptosis), and two (3.4%) had IgG4-ROD. Upper lid retraction was observed in both TED (79.5%) and IOM without ptosis (90.0%). SR tendon involvement was significantly less common in TED than in IOM (13.3% vs. 78.6%, p < 0.001). Infraduction limitation was more prevalent in TED (94.7%) and IOM with ptosis (100%) than in IOM without ptosis (25.0%), while supraduction limitation was noted in IOM without ptosis (75.0%). Compared to IOM without ptosis, IOM with ptosis had a shorter time to diagnosis (0.5 vs. 1.5 months, p = 0.088) and better clinical outcomes following steroid treatment (lid level asymmetry: 0% vs. 50.0%, p = 0.044).

**Conclusion:**

TED and IOM are the most common etiologies of orbital inflammation with isolated SR-LC enlargement. IOM can manifest as either ptosis or retraction, with the latter distinguishable from TED by clinicoradiological features. Early initiation of systemic steroids can be associated with favorable clinical responses, especially for suspected IOM cases; however, further prospective studies are warranted.

## Introduction

Extraocular muscle (EOM) enlargement is most commonly associated with thyroid eye disease (TED), followed by idiopathic orbital myositis (IOM), vascular disease, and neoplastic disease [1–3]. The differential diagnosis can be narrowed down by clinical features and ancillary tests, including orbital imaging studies [1,4]. Given its possible complications, an EOM biopsy is selectively indicated for cases with atypical or inconclusive clinical and radiological findings [3,5].

The superior rectus (SR) and levator palpebrae superioris are closely related in anatomical position and innervation, and are collectively called the superior rectus-levator complex (SR-LC) [6]. In recent studies of TED, SR-LC enlargement has been commonly reported, especially in patients with upper lid retraction [7–9]. Isolated SR-LC enlargement is the initial presentation in as many as 30% of patients with TED [7]. However, isolated SR-LC enlargement is not specific to TED and may also represent other inflammatory or infiltrative diseases, including IOM [10–14].

The differential diagnosis of EOM enlargement is important because of the different diagnostic approaches, systemic involvements, treatment protocols and outcomes. Despite these implications, comparative data focusing specifically on isolated SR-LC enlargement as an initial manifestation of orbital inflammation are limited. This study aimed to analyze the clinical features, radiologic findings, and treatment outcomes of a large number of patients with isolated SR-LC enlargement and to identify findings that may facilitate early and accurate differentiation among underlying etiologies.

## Methods

We retrospectively reviewed the medical records of patients treated for isolated SR-LC enlargement, identified in contrast-enhanced orbital imaging studies at Asan Medical Center from January 2013 to December 2022. We designed the study in March 2022, and data were accessed for research purposes between May 24, 2022 and May 23, 2023. All patients underwent orbital imaging for evaluation of symptoms and signs associated with orbital inflammation, including eyelid retraction/ptosis, eyelid swelling, ocular duction limitation, conjunctival injection/chemosis, and proptosis. Ophthalmologic examination and blood tests for thyroid hormones and antibodies were performed on all patients. A biopsy was performed to exclude a tumorous condition in cases demonstrating heterogeneous enhancement on orbital imaging or diffusion restriction on diffusion-weighted magnetic resonance imaging (MRI) [15]. Patients were followed up for more than 3 months after treatment. Exclusion criteria included delayed orbital imaging studies (> 6 months after initial presentation) and insufficient post-treatment follow-up (< 3 months). The study was conducted according to the tenets of the Declaration of Helsinki. The protocol was approved by our institutional review board (approval No. 2022−0712). Written informed consent for publication of the patient’s clinical information and images was obtained from the patient.

Ophthalmologic examinations were performed by two oculoplastic surgeons (H-SS and MKY). Upper eyelid position was evaluated using margin-reflex distance 1 (MRD1) in the primary position, with normative data in the Korean population indicating a MRD1 of 4 mm [16]. Upper eyelid retraction was defined as a MRD1 of ≥ 6 mm or ≥ 2 mm higher than the fellow eye. Upper eyelid ptosis was defined as a MRD1 of ≤ 2 mm or ≥ 2 mm lower than the fellow eye. Proptosis (> 21 mm or asymmetry > 2 mm) was measured with the Hertel ophthalmometer. Ocular duction limitation and central diplopia within 30 degrees were evaluated with the Hess screen and Goldmann perimetry diplopia tests. Routine blood tests included C-reactive protein, thyroid function test (TFT), thyrotropin binding inhibitor immunoglobulin (TBII, normal range: < 2.0 IU/L) for assessment of thyroid stimulating hormone receptor antibody, and thyroid stimulating immunoglobulin (TSI). According to the manufacturer’s assay protocol, TSI activity is reported as a relative percentage compared with a reference control, with values < 140% considered within the normal range. When routine blood tests are all within the normal range, serum angiotensin converting enzyme (ACE, normal range: < 53 U/L) and immunoglobulin G4 (IgG4, normal range: < 1.35 g/L) were additionally measured.

This study aimed to compare the clinical features and radiological findings among different diagnoses in patients with isolated SR-LC enlargement. To ensure an objective comparison and avoid circular reasoning, we classified the groups based on longitudinal serological data rather than their clinical presentations. TED was defined as an increase in TBII or TSI at any point during the clinical course, and further classified into dysthyroid or euthyroid depending on whether the TFT finally became abnormal. IOM was considered in patients with normal results of blood tests performed three or more times at 3-month intervals, along with the absence of systemic and ophthalmic signs of autoimmune disease (e.g., uveitis/retinitis). Patients with IOM were further divided into subgroups according to the presence or absence of upper lid ptosis.

Once the diagnosis was established strictly by these serological profiles, we compared the clinical features (e.g., eyelid position, ocular duction) and radiologic findings (e.g., tendon involvement, adjacent infiltration) as the primary study outcomes. Time to diagnosis was defined as the time taken from the patient’s awareness of symptoms until a definitive diagnosis was made at our institution.

Coronal orbital computed tomography (CT) and/or MRI images were analyzed for EOM enlargement and perimuscular inflammatory signs. For MRI, evaluations were based on an integrated assessment of standard sequences, including contrast-enhanced T1-weighted images with fat suppression, T2-weighted images, and diffusion-weighted imaging, as available. Muscle thickness was defined as the length of a line perpendicular to the long axis, measured on the image showing the maximum cross-sectional thickness of the SR–LC [17]. The isolated enlargement of SR-LC was defined as an SR–LC thickness ≥ the mean + 2 standard deviations (SDs) (≈ 5.5 mm in the Korean population [18]) of normal SR–LC thickness, with no other EOMs meeting this criterion. In the same section, the SR muscle was carefully traced, and SR enlargement was defined as an SR thickness ≥ mean + 2 SDs (≈ 3.0 mm in the Asian population [17]) of normal SR thickness. A ratio of SR tendon width measured at the globe equator to SR belly width measured at the midpoint greater than 0.5 was considered SR tendon involvement [19]. Contrast-enhanced perimuscular infiltrations of the adjacent orbital fat (i.e., blurring of the SR-LC margin), orbital roof, and superotemporal intermuscular septum [20] were counted. The superior ophthalmic vein (SOV) diameter in our study was measured on retrobulbar coronal images along its intraorbital course, and the largest measured diameter was used for analysis; an SOV diameter ≥ 2 mm was considered as SOV enlargement [21].

All patients were initially treated with orally- or intravenously-administered systemic corticosteroids. Severity of TED was assessed according to the 2016 EUGOGO classification [22]. Patients with moderate to severe TED were treated with intravenous methylprednisolone (IV mPd) administered once a week for 12 weeks (500 mg weekly for six weeks, then 250 mg weekly for six weeks) [22]. For patients with mild TED or orbital inflammation other than TED, oral prednisolone (Pd) was given at an initial dose of 0.6 mg/kg/day for one week and then tapered by 5 mg every 2 weeks. Post-treatment clinical signs were evaluated at 3 months after termination of systemic steroids. If MRD1 asymmetry or ocular duction limitation did not improve despite systemic treatment, local triamcinolone (TA) injections were performed in the deep superior intraorbital area at a dose of 0.5 mL of a 40 mg/mL solution. If ocular duction limitation did not respond to up to two TA injections administered at one-month intervals, additional radiation therapy (20 Gy in 10 fractions) was subsequently administered. Follow-up imaging studies were performed at 3 months after these treatment or when a relapse was suspected. Radiologic response was classified as ‘worsened’ if the maximal SR-LC area increased, ‘stationary’ if it decreased by 0–25%, ‘partially improved’ if it decreased by 25–75% but remained larger than the contralateral eye, and ‘completely improved’ if there was no noticeable difference. Orbital decompression, SR recession, and levator recession were considered for residual proptosis, vertical strabismus, and upper lid retraction despite steroid treatments and radiation therapy.

Statistical analyses were performed using IBM SPSS Statistics software (version 21.0, IBM Corp., Armonk, NY, USA). The Mann–Whitney U test was used to compare continuous variables, and Fisher’s exact test was used to compare categorical variables. A two-sided p-value < 0.05 was considered statistically significant.

## Results

Sixty-four eyes of 58 patients were included in this study, with a median age of 48.1 years (interquartile range [IQR], 36.4–56.3). TED was diagnosed in 39 (67.2%) patients (median TSI level: 340%, range: 144–945), including 16 patients (41.0%) who remained euthyroid throughout the follow-up. Among the 23 dysthyroid patients, 22 were hyperthyroid and 1 was hypothyroid, and all patients were referred to an endocrinologist for appropriate management. IOM was diagnosed in 17 (29.3%) patients (median TSI level 55.4%, range: 27–106). The median age at diagnosis showed no significant difference between the TED and IOM groups (47.7 years vs. 44.3 years, p = 0.650, Mann-Whitney U test). Two patients (3.4%) were diagnosed with IgG4-related ophthalmic disease (IgG4-ROD) based on the results of lacrimal gland biopsy and elevated serum IgG4, but they were not included in the comparative analyses due to the small number.

Baseline characteristics, such as demographics, clinical signs, and radiological findings of all patients according to the diagnosis are presented in Table 1. The median age at diagnosis showed no significant difference between the TED and IOM groups (47.7 years vs. 44.3 years, p = 0.650, Mann-Whitney U test). Patients with TED had a higher proportion of women (71.8% vs. 41.2%, p = 0.039, Fisher’s exact test) and were diagnosed later (2.8 months vs. 0.8 months, p < 0.001) compared to patients with IOM. Patients with TED also had less frequent tendon-involving SR enlargement than patients with IOM (13.3% vs 78.6%, p < 0.001).

**Table 1:** Baseline characteristics of patients with isolated superior rectus-levator complex (SR-LC) enlargement.

n = 58	TED (n = 39)	IOM (n = 17)	*p*-value^a^	IgG4-ROD (n = 2)
Female sex, n (%)	28 (72)	7 (41)	**0.039**	2 (100)
Time to diagnosis, months (IQR)	2.8 (1.7‒5.8)	0.8 (0.4‒2.0)	**< 0.001**	5.3 (1.4‒9.2)
Age at diagnosis, years (IQR)	47.7 (35.4‒55.4)	44.3 (35.7‒55.5)	0.650	64.5 (60.0‒69.1)
Clinical signs, n (%)				
Bilaterality	6 (15)	0 (0)	0.163	0 (0)
Upper lid ptosis (> 2 mm)	0 (0)	7 (41)	**< 0.001**	0 (0)
Upper lid retraction (> 2 mm)	31 (79)	9 (53)	0.058	1 (50)
Lid lag or lagopthalmos	29 (74)	8 (47)	0.067	0 (0)
Eyelid swelling	32 (82)	14 (82)	> 0.99	2 (100)
Proptosis (> 2 mm)	21 (54)	8 (47)	0.773	1 (50)
Ocular duction limitation, n (%)				
On 9 gaze photography	12 (31)	3 (18)	0.513	0 (0)
On Hess screen test	19 (49)	8 (47)	> 0.99	0 (0)
Central (≤ 30°) diplopia, n (%)	11 (28)	4 (24)	> 0.99	0 (0)
Radiological findings, n (%)				
SR-LC enlargement				
SR enlargement	30 (77)	14 (82)	> 0.99	1 (50)
SR tendon involvement	4/30 (13)	11/14 (79)	**< 0.001**	1/1 (100)

IgG4-ROD, immunoglobulin G4-related ophthalmic disease; IOM, idiopathic orbital myositis; IQR, interquartile range; TED, thyroid eye disease.

Continuous variables are presented as medians (IQR), and values in bold indicate statistical significance.

^a^Comparison of TED and IOM groups using the Fisher’s exact test or Mann‒Whitney U test.

**Fig 1:**
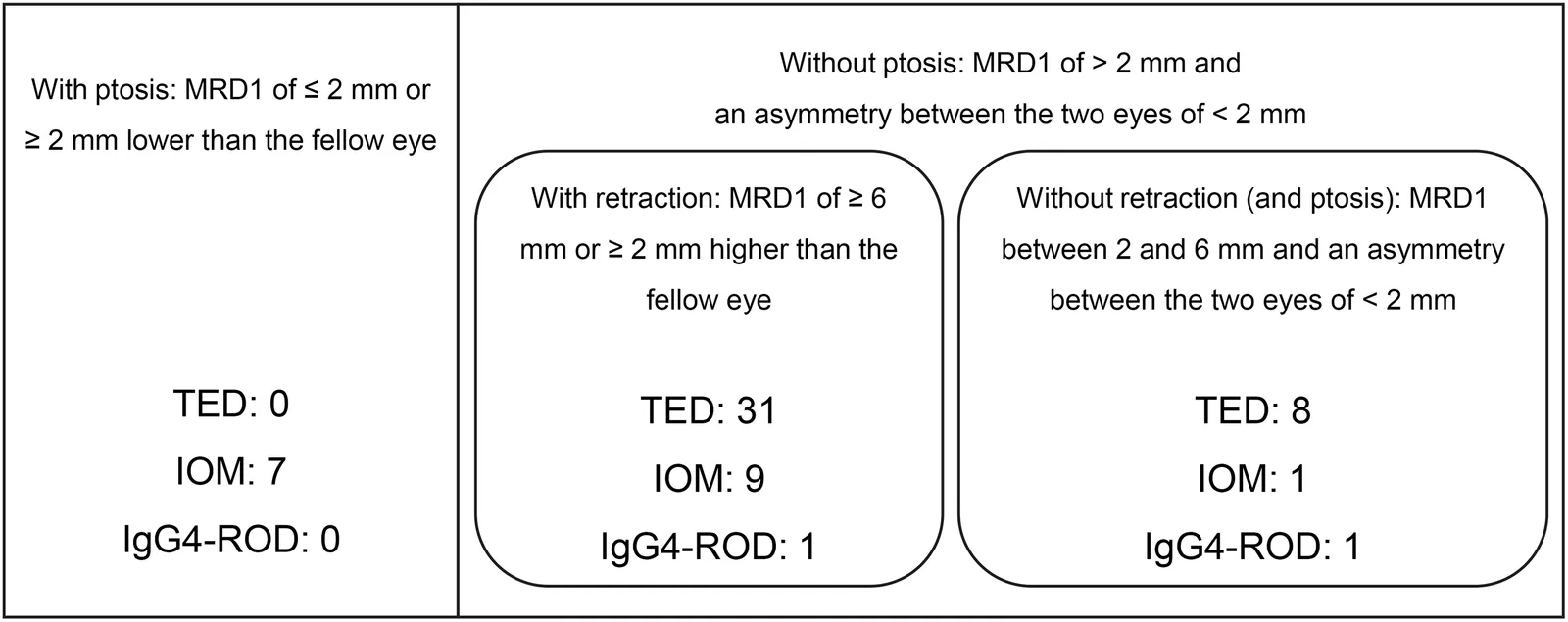
Definition of upper eyelid ptosis or retraction based on margin reflex distance 1 (MRD1), and the distribution of thyroid eye disease (TED), idiopathic orbital myositis (IOM), and immunoglobulin G4-related ophthalmic disease (IgG4-ROD) across each category.

Upper lid ptosis was observed in some patients with IOM but not in those with TED (41.2% vs. 0%, p < 0.001), allowing for subgrouping of IOM (Fig 1). Since ptosis was unique to IOM and absent in TED, we did not compare the IOM with ptosis group with the TED group, as ptosis clearly differentiated them. Instead, we compared IOM without ptosis, which can resemble TED due to upper lid retraction, to TED. Additionally, we compared the IOM without ptosis group with the IOM with ptosis group. These comparisons are shown in Table 2.

**Table 2:** Baseline characteristics of patients with TED and IOM with or without upper lid ptosis.

	TED (n = 39)	IOM without upper lid ptosis (n = 10)	IOM with upper lid ptosis (n = 7)	*p*-value^a^	*p*-value^b^
Female sex, n (%)	28 (72)	3 (30)	4 (57)	**0.026**	0.350
Time to diagnosis, months (IQR)	2.8 (1.7‒5.8)	1.5 (0.6‒3.7)	0.5 (0.4‒1.0)	0.053	0.088
Clinical signs, n (%)					
Bilaterality.	6 (15)	0 (0)	0 (0)	0.324	> 0.99
Upper lid retraction (> 2 mm)	31 (79)	9 (90)	0 (0)	0.663	**< 0.001**
Lid lag or lagopthalmos	29 (74)	8 (80)	0 (0)	> 0.99	**0.002**
Eyelid swelling	32 (82)	7 (70)	7 (100)	0.405	0.228
Proptosis (> 2 mm)	21 (54)	4 (40)	4 (57)	0.496	0.637
Ocular duction limitation, n (%)					
On 9 gaze photography	12 (31)	1 (10)	2 (29)	0.253	0.537
On Hess screen test	19 (49)	4 (40)	4 (57)	0.731	0.637
Supraduction limitation	2/19 (11)	3/4 (75)	1/4 (25)	**0.021**	0.486
Infraduction limitation	18/19 (95)	1/4 (25)	4/4 (100)	**0.009**	0.143
Central (≤ 30°) diplopia, n (%)	11 (28)	3 (30)	1 (14)	> 0.99	0.603
Radiological findings, n (%)					
SR-LC enlargement					
SR enlargement	30 (77)	8 (80)	6 (86)	> 0.99	0.603
SR tendon involvement	4/30 (13)	7/8 (88)	4/6 (67)	**< 0.001**	> 0.539
Adjacent infiltration					
Orbital fat	7 (18)	0 (0)	6 (86)	0.319	**< 0.001**
Orbital roof	9 (23)	1 (10)	3 (43)	0.663	0.250
SOV enlargement	11 (28)	0 (0)	3 (43)	0.090	0.052
Superotemporal intermuscular septum	30 (77)	3 (30)	4 (57)	**0.009**	0.350

IgG4-ROD, immunoglobulin G4-related ophthalmic disease; IOM, idiopathic orbital myositis; IQR, interquartile range; SOV, superior ophthalmic vein; TED, thyroid eye disease.

Continuous variables are presented as medians (IQR), and values in bold indicate statistical significance.

^a^Comparison of TED and IOM without ptosis groups using the Fisher’s exact test or Mann‒Whitney U test.

^b^Comparison of IOM with and without ptosis groups using the Fisher’s exact test or Mann‒Whitney U test.

Most clinical characteristics overlapped between the TED and IOM without ptosis groups, with upper lid retraction observed in 79.5% of patients with TED and 90.0% of patients with IOM without ptosis (Table 2). The main difference was the direction of ocular duction limitation: infraduction limitation was more common in TED than in IOM without ptosis (94.7% vs. 25.0%, p = 0.009), while supraduction limitation was more common in IOM without ptosis than in TED (75.0% vs. 10.5%, p = 0.021). Additionally, adjacent infiltration on orbital imaging was more common in TED than in IOM without ptosis, with a significant difference observed in the superotemporal intermuscular septum (76.9% vs. 30.0%, p = 0.009).

**Fig 2:**
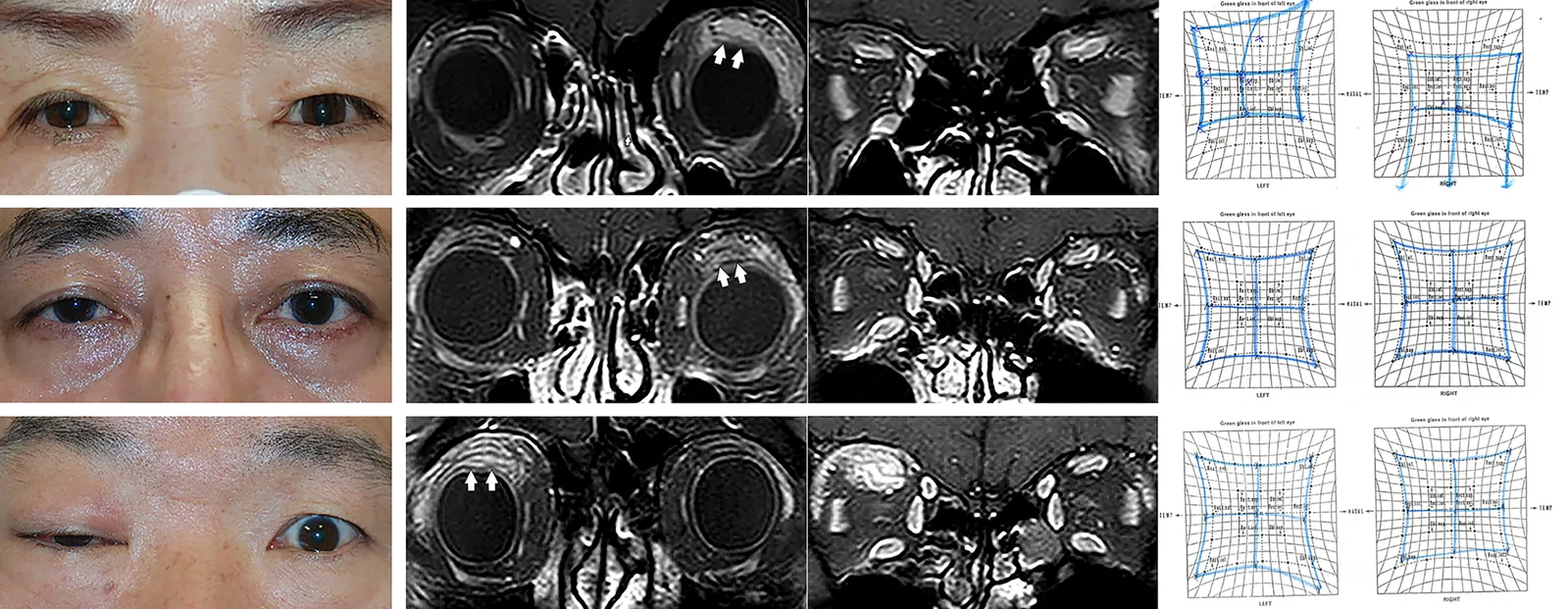
Representative cases with isolated superior rectus-levator complex (SR-LC) enlargement. The magnetic resonance images are sharpened coronal sections at the SR tendon (arrow) insertion site and muscle belly. (Top row) Thyroid eye disease manifested as upper lid retraction, tendon-sparing SR enlargement, and restricted infraduction of the left eye. (Middle row) Idiopathic orbital myositis (IOM) with upper lid retraction manifested as tendon-involving SR enlargement with minimal adjacent infiltration and restricted supraduction of the left eye. (Bottom row) IOM with upper lid ptosis manifested as tendon-involving SR enlargement with marked infiltration into the adjacent orbital wall and intermuscular septum, and restricted infraduction of the right eye.

IOM with ptosis had a shorter median time to diagnosis compared to IOM without ptosis (0.5 vs. 1.5 months), although this difference was not statistically significant (p = 0.088) (Table 2). Compared to IOM with ptosis, IOM without ptosis presented more frequent upper lid retraction (90.0% vs. 0%, p < 0.001). Lid lag or lagophthalmos was also more common in IOM without ptosis than in IOM with ptosis (80.0% vs 0%, p = 0.002). Additionally, supraduction limitation was more common in IOM without ptosis (75.0% vs 25.0%), while infraduction limitation was more prevalent in IOM with ptosis (100% vs 25.0%), although this difference was not statistically significant (p > 0.05). Radiological findings revealed that infiltration into the adjacent orbital fat was significantly more common in IOM with ptosis than in IOM without ptosis (85.7% vs 0%, p < 0.001). Representative cases of TED, IOM with retraction, and IOM with ptosis are shown in Fig 2.

Patients were followed for a median duration of 12.6 months (IQR, 6.4‒27.5) after treatment. The mainstay of systemic corticosteroid administration was IV mPD for TED (61.6%) and oral Pd for IOM (94.1%). Residual ocular duction limitation, measured by the Hess screen test, was observed in 35.9% of patients with TED and 5.9% of those with IOM (p = 0.023). Follow-up imaging at approximately 1 year (14.4 months in TED vs. 11.7 months in IOM, p = 0.584) showed that worsening of SR-LC enlargement in 12.0% of patients with TED and 0% of those with IOM. New enlargement of other EOMs was observed in 52.0% of patients with TED and 20% of those with IOM. These differences were not statistically significant (all p > 0.05). Additional radiation therapy was required in some patients with TED (17.9%), but in none of the patients with IOM (p = 0.088).

Treatments and outcomes of each group are presented in Table 3. In all IOM patients with ptosis (n = 7), clinical signs and ocular duction limitations resolved, SR-LC enlargement improved, and there was no new EOM enlargement in follow-up imaging studies after oral Pd treatment. In contrast, IOM patients without ptosis (n = 10) showed less improvement in clinical signs, ocular duction limitations, and SR-LC enlargement. Upper lid level (MRD1) asymmetry completely improved in IOM patients with ptosis but persisted in half of IOM patients without ptosis (improvement rate: 100% vs. 50.0%, p = 0.044).

**Table 3:** Treatments and outcomes of patients with isolated superior rectus-levator complex (SR-LC) enlargement.

n = 58	TED (n = 39)	IOM without upper lid ptosis (n = 10)	IOM with upper lid ptosis (n = 7)	*p*-value^a^	*p*-value^b^	IgG4-ROD (n = 2)
Follow-up duration, months (IQR)	15.2 (8.5‒34.7)	7.1 (3.9‒18.9)	3.2 (2.8‒12.2)	0.140	0.315	55.2 (30.2‒80.2)
Systemic steroid treatment, n (%)						
Oral prednisolone	13 (33)	9 (90)	7 (100)			1 (1/2)
IV methylprednisolone	24 (62)	1 (10)	0 (0)			1 (1/2)
Number of TA injections, n (%)	14 (36)	3 (30)	0 (0)	> 0.99	0.228	0 (0)
Outcomes after steroid treatment						
Residual signs, n (%)						
MRD1 asymmetry > 2 mm (retraction or ptosis)	13 (33)	5 (50)	0 (0)	0.465	**0.044**	0 (0)
Eyelid swelling	4 (10)	3 (30)	0 (0)	0.140	0.228	0 (0)
Proptosis (> 2 mm)	11 (28)	3 (30)	2 (29)	> 0.99	> 0.99	0 (0)
Residual duction limitation, n (%)						
On 9 gaze photography	9 (23)	1 (10)	0 (0)	0.663	> 0.99	0 (0)
On Hess screen test	14 (36)	1 (10)	0 (0)	0.464	0.485	0 (0)
Residual central (≤ 30°) diplopia	4 (10)	1 (10)	0 (0)	> 0.99	> 0.99	0 (0)
Radiological findings, n	25	8	2			2
SR-LC enlargement, n (%)						
Worsened	3 (12)	0 (0)	0 (0)	> 0.99	> 0.99	0 (0)
Stationary	6 (24)	2 (25)	0 (0)	> 0.99	> 0.99	0 (0)
Improved (partial)	14 (56)	5 (68)	1 (50)	> 0.99	> 0.99	2 (2/2)
Improved (complete)	2 (8)	1 (13)	1 (50)	> 0.99	0.378	0 (0)
Newly developed lesion, n (%)						
Any extraocular muscle	13 (52)	2 (25)	0 (0)	0.148	> 0.99	1 (1/2)
Inferior rectus	10 (40)	1 (13)	0 (0)	0.129	> 0.99	0 (0)
Medial rectus	9 (36)	2 (25)	0 (0)	0.356	> 0.99	0 (0)
Lateral rectus	0 (0)	1 (13)	0 (0)	> 0.99	> 0.99	1 (1/2)
Superior oblique	1 (4)	0 (0)	0 (0)	> 0.99	> 0.99	0 (0)
Lacrimal gland	17 (68)	4 (50)	2 (100)	0.420	0.467	2 (2/2)
Bilaterality	9 (36)	1 (13)	0 (0)	0.382	> 0.99	1 (1/2)
Treatment beyond corticosteroids, n (%)						
Radiation therapy	7 (18)	0 (0)	0 (0)	0.319	> 0.99	0 (0)
Surgery						
Orbital decompression	2 (5)	0 (0)	0 (0)	> 0.99	> 0.99	0 (0)
SR recession	2 (5)	0 (0)	0 (0)	> 0.99	> 0.99	0 (0)
Levator surgery	4 (10)	0 (0)	0 (0)	0.569	> 0.99	0 (0)

IgG4-ROD, immunoglobulin G4-related ophthalmic disease; IOM, idiopathic orbital myositis; IQR, interquartile range; MRD1, margin reflex distance 1; TA, triamcinolone; TED, thyroid eye disease.

Continuous variables are presented as medians (IQR), and values in bold indicate statistical significance.

^a^Comparison of TED and IOM without upper lid ptosis groups using the Fisher’s exact test or Mann‒Whitney U test.

^b^Comparison of IOM with and without upper lid ptosis groups using the Fisher’s exact test or Mann‒Whitney U test.

## Discussion

In this study, we found that TED (67%) and IOM (29%) were the most common etiologies of isolated SR-LC enlargement. Compared to IOM, TED had a higher proportion of female patients and was diagnosed later after symptom onset. Upper lid ptosis, observed in 41% of patients with IOM, was exclusive to IOM. Upper lid retraction was common in both TED (79%) and IOM without ptosis (90%). Infraduction limitation was more frequent in TED (95%) and IOM with ptosis (100%), while supraduction limitation predominated in IOM without ptosis (75%). Radiologically, SR tendon involvement was common in IOM (79%), and adjacent infiltration was infrequent in IOM without ptosis.

The rate of euthyroidism among patients with TED in our study was 41.0%, which was significantly higher than the rate (14.7%) reported in a previous study of Korean patients with TED [23]. Similarly, in the United States [9,24], the rate of euthyroidism was higher in patients with TED presenting with SR-LC enlargement than in the overall TED cohort. Euthyroid TED is known to be clinically less active and severe than hyperthyroid TED [23,25]. Levels of TBII and TSI correlated with the number of involved EOMs and were higher in hyperthyroid TED than in euthyroid TED [23,25]. Therefore, the higher proportion of patients with euthyroid TED may reflect the characteristics of isolated SR-LC enlargement as a single EOM involvement.

In our study, IOM with upper lid retraction comprised a significant portion of IOM (53%, 9/17), a finding rarely reported in previous studies on IOM [26]. Elevated TSI was crucial in distinguishing between IOM and TED, especially in cases with retraction, as TBII was elevated in only about one-third of patients with euthyroid TED. Kazuo et al. [27] emphasized the usefulness of repeated TSI assessments in patients with proptosis or lid retraction, as TSI measurement can yield false negative results in early or stable TED. We excluded patients diagnosed more than 6 months after symptom onset to avoid false negative TSI values due to its gradual decrease [28] and remeasured TSI at 3-month intervals in patients with IOM to detect patients with TED with late TSI increase.

We identified distinct patterns of ocular duction limitation and upper lid levels among patients with TED, IOM without ptosis, and IOM with ptosis, suggesting that clinical presentations may vary depending on the etiology and phase of EOM inflammation. Upper lid retraction in orbital inflammation may arise from sympathetic overactivity, restrictive fibrosis of the levator or Müller’s muscle, proptosis, and weakened orbicularis tone [29], while ptosis may result from levator weakness or mass effects from the swelling [3]. Siatkowski et al. [30] claimed that the muscle dysfunction in orbital myositis progresses from an early inflammatory phase with muscle weakness in the first two weeks to a later restrictive phase with muscle fibrosis. In this context, patients with TED often present with infraduction limitation and lid retraction, suggesting that EOM inflammation in TED may involve fibrosis even in the early phases. In contrast, IOM was divided into two subgroups: IOM with ptosis (41%), where inflammatory swelling and weakness are more prominent in the early phase, and IOM without ptosis (59%), where fibrotic retraction and restriction play a larger role in the later stage.

IOM with ptosis had a shorter median time to diagnosis (2 weeks) than IOM without ptosis (6 weeks), suggesting that ptosis and retraction may reflect an weak-to-restrictive phase shift in SR-LC involvement. In IOM with ptosis, lid ptosis can be attributed to levator muscle swelling and weakness, while infraduction limitation might result from inflammatory swelling around the SR. In IOM without ptosis, progressive fibrosis in the levator muscle leads to retraction. Supraduction limitation might result from sustained weakness or tendon-involving enlargement of SR [19]. Further studies on the longitudinal changes n the upper lid signs, SR-LC size, and enhancement in patients with untreated IOM would be valuable to validate this hypothesis.

Radiological findings and treatment outcomes also differed among the groups. As reported previously [3,9,31], TED was more likely to show initial tendon-sparing SR enlargement, bilateral involvement, worsening of SR-LC enlargement, or additional enlargement of other EOMs during follow-up compared to IOM. We found more favorable outcomes following systemic steroids in IOM than TED regarding the ocular duction and lid levels, consistent with the results of a previous study [32]. IOM has a more acute onset compared to TED [33] and can cause greater discomfort in daily life as it may be accompanied by ptosis. These characteristics of IOM may help shorten the time to diagnosis and treatment, prevent EOM fibrosis, and improve steroid responsiveness [26,33]. These findings may suggest the need for more aggressive and prolonged treatments, such as high-dose IV steroids, and extended follow-up with imaging in TED. In the IOM subgroups, IOM without ptosis showed less adjacent infiltration and a smaller maximal area of SR-LC than IOM with ptosis, supporting the hypothesis that ptosis is linked to acute inflammation, while retraction is associated with fibrosis [10]. Clinical improvement in retraction occurred in only about half of the patients with IOM following treatment with systemic steroids, indicating that more invasive treatments, such as TA injection and levator recession surgery, may be eventually required in IOM without ptosis. Our findings highlight that early detection and timely treatment during the acute ptotic phase, prior to progression to the later retractive phase, may improve the outcomes in patients with IOM involving the SR-LC.

Recent studies in Asian populations have suggested that orbital imaging may identify progressive or vision-threatening TED that is underestimated by conventional assessment using the Clinical Activity Score (CAS) [34,35]. Furthermore, comprehensive reviews have highlighted the expanding role of orbital imaging in TED, emphasizing quantitative imaging and artificial intelligence-assisted image analysis of orbital structures as objective imaging biomarkers for diagnosis, activity assessment, prediction of dysthyroid optic neuropathy, and treatment response [36,37]. In this context, we did not stratify patients with TED according to CAS because our primary objective was to compare the clinical and imaging characteristics of isolated SR-LC enlargement between TED and IOM rather than disease activity within TED. Our findings suggest that isolated SR-LC enlargement, a previously underrecognized imaging feature, represents a distinct imaging manifestation with clinically relevant diagnostic implications.

In addition to TED and IOM, a broad spectrum of diseases can cause enlargement of the SR-LC, including sarcoma, metastatic disease, hematologic malignancies such as lymphoma or leukemia. Some case reports have suggested that sarcoidosis or IgG4-ROD may be potential causes of upper lid retraction [38,39]. A definitive diagnosis in these cases was made via pathologic examination of tissues obtained during levator recession or from the mass-like SR enlargement. However, when SR-LC enlargement is mild and non-mass-forming, as in our patients, biopsy may be challenging due to risks of complications like strabismus and ptosis [5]. Therefore, in patients with upper lid retraction and normal TSI, additional serologic tests should be considered to identify other specific diseases. While oral Pd may also yield good clinical and radiological responses in patients with IgG4-ROD or sarcoidosis [40,41], there are differences in the need for systemic work-up and prognosis compared to IOM.

There are some limitations of our study. Specific causes of orbital inflammation were not completely ruled out because biopsy was rarely performed. However, we conducted meticulous serologic tests, repeated TSI assessments, and radiological evaluations to enhance the diagnostic accuracy. Both TED and IOM are known to commonly exhibit fibroblast infiltration with mild fibrosis, and lymphocytic infiltration, while lacking dense lymphoid aggregates or follicle formation on biopsy in the early stages [42–44]. Accordingly, early SR–LC biopsy is considered to have limited clinical utility in differentiating between these two entities. Secondly, the follow-up duration was relatively shorter for patients with IOM compared to those with TED, likely because some patients with TED experienced disease progression involving other EOMs. Furthermore, half of the IOM patients with ptosis did not undergo post-treatment imaging, limiting radiological comparisons. This is primarily attributable to the better response to initial steroid treatment in IOM patients with ptosis, which reduced the need for follow-up imaging and led to higher follow-up loss in this group. Lastly, our study included a small number of IgG4-ROD cases. Since these cases were pathologically confirmed, they needed to be presented separately from IOM. They were excluded from statistical analysis to focus on the comparison between IOM and TED.

In conclusion, for patients with upper lid retraction or ptosis, lid swelling, or diplopia, prompt orbital imaging and blood tests, including TFT and TSI, may be helpful in evaluating potential SR-LC involvement and the underlying causes. In our study, TED and IOM are the most common etiologies, suggesting a potential role for repeated TSI measurements. IOM may present with either upper lid ptosis or retraction, and distinguishing the latter from TED may be facilitated by specific ocular duction limitation patterns, adjacent tissue infiltration, and SR tendon involvement. Early initiation of systemic steroids can be associated with favorable clinical responses, particularly in suspected cases of IOM; however, further prospective studies are warranted to establish definitive treatment recommendations.
